# Application of the Analytical Hierarchy Process in the management of private ambulance care systems in three selected European countries: a strategic decision-making framework

**DOI:** 10.3389/fpubh.2025.1526586

**Published:** 2025-05-06

**Authors:** Jalal Rezaei, Roya Azouji, Mina Azouji, Hamzeh Ghorbani

**Affiliations:** ^1^Department of Medical Surgical Nursing, Shahid Beheshti University of Medical Sciences, Tehran, Iran; ^2^Department of Emergency Nursing, School of Nursing and Midwifery, Tehran University of Medical Sciences, Tehran, Iran; ^3^KfH Kuratorium für Dialyse und Nierentransplantation, Dortmund, Germany; ^4^Young Researchers and Elite Club, Ahvaz Branch, Islamic Azad University, Ahvaz, Iran; ^5^Faculty of General Medicine, University of Traditional Medicine of Armenia (UTMA), Yerevan, Armenia

**Keywords:** Analytical Hierarchy Process (AHP), private ambulance services, regulatory compliance, service quality, resource allocation

## Abstract

Private ambulance services play a vital role in healthcare systems across Europe, supplementing public emergency services and providing essential medical transportation. However, managing these services presents significant challenges, including resource allocation, regulatory compliance, service quality, technological integration, workforce management, and financial sustainability. This study employs the Analytical Hierarchy Process (AHP) as a strategic decision-making tool to optimize the management of private ambulance services in Germany, Spain, and the United Kingdom. To achieve this, data were collected from 20 participants across the three countries (Germany: 7, Spain: 6, United Kingdom: 7), comprising ambulance service administrators, emergency medical personnel, and regulatory experts. A purposive sampling method was used to ensure the inclusion of key stakeholders with direct experience in the sector. Participants completed structured questionnaires involving pairwise comparisons of key decision criteria. Results reveal that Regulatory Compliance is the highest priority across all countries (Germany: 0.25, Spain: 0.22, UK: 0.20), followed by Service Quality, which is particularly emphasized in the UK (0.22) and Germany (0.20). Technological Integration is important in Spain (0.20), reflecting the need for advancements in underserved areas. While Workforce Management and Financial Sustainability rank slightly lower, they remain critical for operational efficiency. The study highlights country-specific challenges and regulatory differences and provides actionable recommendations for optimizing resource allocation, improving service quality, and ensuring compliance. Despite limitations such as potential biases and a narrow geographic focus, the findings offer valuable insights for refining management practices and enhancing the sustainability of private ambulance services across Europe.

## 1 Introduction

Private ambulance services in Europe play a critical role in the healthcare system, providing essential medical transportation and emergency care across diverse geographic regions and healthcare environments (1, 2). These services operate alongside public emergency services, often filling gaps in coverage and offering specialized transport options for non-emergency situations, inter-hospital transfers, and patient repatriation (1, 3, 4). The landscape of private ambulance services varies significantly across European countries and is influenced by differing healthcare policies, regulatory frameworks, and funding mechanisms. In some regions, private providers are integrated into the national emergency response system, while in others, they function more independently, catering to specific markets or regions (5, 6). The importance of efficient management in this sector cannot be overstated, as it directly impacts the quality of care provided to patients, response times, and the overall cost-effectiveness of healthcare delivery. Efficient management involves optimizing resource allocation, ensuring compliance with stringent regulatory standards, and maintaining high levels of service quality, all crucial for meeting patients' diverse and often urgent needs. Furthermore, navigating the complex regulatory landscape and managing operational challenges such as staffing, fleet maintenance, and technological integration is essential for the sustainability and growth of European private ambulance services (7, 8). As demand for these services continues to rise, driven by an aging population and increasing healthcare needs, the pressure on private ambulance providers to deliver reliable, timely, and cost-effective care is more significant than ever.

**Figure 1:**
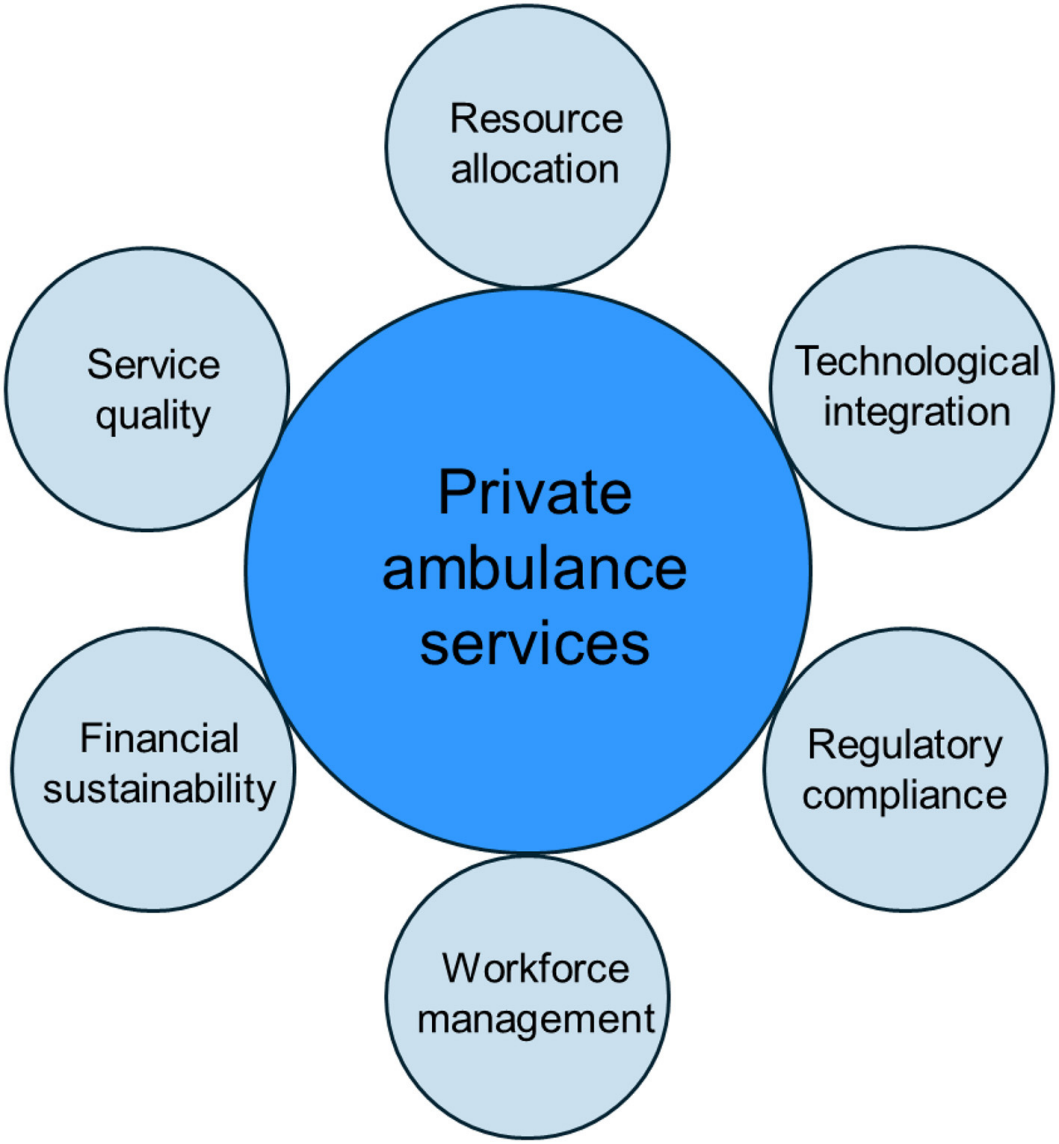
Key operational and strategic challenges affecting private ambulance services include resource allocation, service quality, regulatory compliance, technological integration, workforce management, and financial sustainability.

Managing private ambulance services in Europe involves navigating complex challenges that require strategic planning and effective decision-making (9, 10). *Resource allocation* remains one of the foremost challenges, where operators must juggle the efficient distribution of vehicles, medical equipment, and personnel across various emergency and non-emergency situations. This challenge is heightened by balancing cost-efficiency with delivering high-quality care, often within tight budget constraints and a highly competitive market environment (11, 12). Another significant challenge is ensuring *service quality* across diverse geographic regions and healthcare systems. Patients and healthcare providers expect private ambulance services to meet rigorous standards, including rapid response times, the availability of advanced medical equipment, and highly trained medical personnel. Achieving and maintaining these standards is difficult, especially in rural or underserved areas where resources are more limited, potentially leading to inconsistencies in the level of care provided (13, 14). The main challenges of private ambulance services are schematically shown in Figure 1.

*Regulatory compliance* is a further layer of complexity, as the regulatory landscape for ambulance services varies widely across Europe. Operators must navigate an intricate web of rules related to licensing, operational standards, safety protocols, and reimbursement policies. Compliance with these regulations is critical for legal operations, maintaining public trust, and avoiding significant penalties, such as fines or the revocation of operating licenses (15, 16). Cross-border operations introduce additional regulatory hurdles, as operators must comply with the different regulations of multiple countries, further complicating management efforts (17, 18). Additionally, the increasing demand for *technological integration* poses a significant challenge. Adopting new technologies, such as GPS tracking, electronic health records, and telemedicine, is essential for improving operational efficiency and patient outcomes. However, integrating these technologies requires substantial investment and expertise, which can be particularly challenging for smaller or less financially robust operators. Moreover, there is the challenge of ensuring that all staff are adequately trained to use these new systems effectively, which can be resource-intensive (19, 20). *Workforce management* is another critical challenge, particularly regarding recruiting, training, and retaining qualified personnel. The high-stress nature of ambulance work and the physical and emotional demands placed on staff often lead to high turnover rates. This situation is exacerbated by a general shortage of healthcare professionals across Europe, making it difficult for private ambulance services to maintain a stable and well-trained workforce. Lastly, financial sustainability remains an ongoing concern. Private ambulance operators often face pressure to keep costs low while meeting high care standards (6, 21). Fluctuations in funding, reimbursement rates, and operational costs, combined with competition from public services and other private providers, make it challenging to maintain financial viability. Investing in new technologies, meeting regulatory requirements, and ensuring high service quality further strains financial resources. These interconnected challenges—resource allocation, service quality, regulatory compliance, technological integration, workforce management, and financial sustainability—create a complex operating environment for European private ambulance services (22–24). To address these challenges effectively, there is a pressing need for robust decision-making frameworks that can help operators optimize their operations, enhance service delivery, and ensure long-term sustainability in a rapidly changing healthcare landscape.

We have reviewed key research on emergency medical services (EMS), the Analytical Hierarchy Process (AHP) application in healthcare, and the challenges in managing private ambulance services. The studies included a wide range of healthcare systems and addressed different aspects of service quality, regulatory compliance, resource allocation, technological integration, workforce management, and financial sustainability. Many of these works align with our study's objectives, particularly optimizing private ambulance service management using AHP-based decision-making frameworks. Table 1 summarizes and categorizes relevant studies, comparing their key findings with our research.

**Table 1:** Comparison of related studies on EMS, AHP applications, and private ambulance service challenges with this paper.

**Category**	**Study**	**Key findings**	**Comparison with this paper**
Ambulance Services in Europe	Fischer et al. (25)	EP-based EMS improves survival rates and patient outcomes due to ALS expertise.	It aligns with our emphasis on service quality, highlighting the importance of skilled personnel in private ambulance services.
	Khaki et al. (26)	AHP applied to EMS location selection; EMS efficiency depends on proximity to cities and intersections.	Supports our use of AHP for resource allocation and optimizing ambulance placement.
	Reuter-Oppermann et al. (27)	EMS logistics vary by country; universal EMS guidelines are challenging.	This reinforces our finding that regulatory compliance differs across Germany, Spain, and the UK, requiring localized strategies.
	Metelmann et al. (28)	Improved EMS integration reduces delays and enhances outcomes.	Supports our findings on regulatory frameworks affecting private ambulance service efficiency.
	Hayes et al. (29)	Safety performance indicators in ambulance procurement vary in interpretation, affecting safety standards.	Aligns with our focus on regulatory compliance as the top priority for private ambulance services.
	Khattak et al. (30)	Patient satisfaction depends on cleanliness and service quality.	Reinforces our emphasis on service quality, especially in UK and Germany, where high standards are critical.
	Fager et al. (31)	Advisory decision support systems (ADSS) improve nurse confidence but create challenges in role adaptation.	Supports our research on technological integration, showing that while new systems improve efficiency, they must align with operational needs.
Applications of AHP in Healthcare	Pecchia et al. (32)	AHP helps prioritize fall prevention risk factors based on clinician input.	It supports our use of AHP in ambulance service management and demonstrates its effectiveness in structured decision-making.
	Schmidt et al. (33)	AHP use in healthcare has increased but lacks a consistent methodology.	Highlights the importance of clear AHP methodology, which our study ensures through systematic pairwise comparisons.
	Sahin et al. (34)	AHP applied to hospital site selection; demand was the most important factor.	Relates to our use of AHP for decision-making in ambulance services, prioritizing regulatory compliance and service quality.
	Singh and Prasher (35)	Combined Fuzzy AHP and SERVQUAL to assess hospital service quality.	Reinforces our study's emphasis on service quality but applied to ambulance operations instead of hospitals.
	Watróbski (36)	SSP-AHP applied to social sustainability in healthcare; Nordic countries score highest.	Supports our financial sustainability criterion but with a broader healthcare system perspective.
	Senapati and Panda (37)	Fuzzy AHP used to rank hospitals based on patient experience (PX).	Similar to our AHP approach, but our focus is on ambulance services rather than hospital settings.
	Pant et al. (38)	AHP applied to smart healthcare decision-making; calm state of mind ranked most critical.	Demonstrates AHP's flexibility, though our study focuses on ambulance management rather than general healthcare decisions.
Challenges in Managing Private Ambulance Services	Gupta et al. (39)	Public-Private Partnership (PPP) model in India improved service availability but had government partnership challenges.	Supports our research, where private ambulance services also face regulatory hurdles, requiring better policy integration.
	Holmberg et al. (40)	EMS managers need holistic patient assessment and disease management knowledge.	Reinforces our focus on workforce management, particularly the importance of trained personnel.
	O'Cathain et al. (41)	Ambulance non-conveyance rates vary based on paramedic role differences and service-level factors.	Highlights regulatory discrepancies, similar to the variation in compliance requirements across our studied countries.
	Kavuma et al. (42)	Financial management in Uganda's public EMS requires alternative funding strategies.	It supports our research on financial sustainability, though our study focuses on private ambulance services.
	Slotsvik et al. (43)	Air ambulance procurement benefits from relational governance, balancing flexibility and stability.	This aligns with our findings that regulatory compliance and operational flexibility are key to sustainable private ambulance management.

## 2 Methodology

### 2.1 Workflow diagram

**Figure 2:**
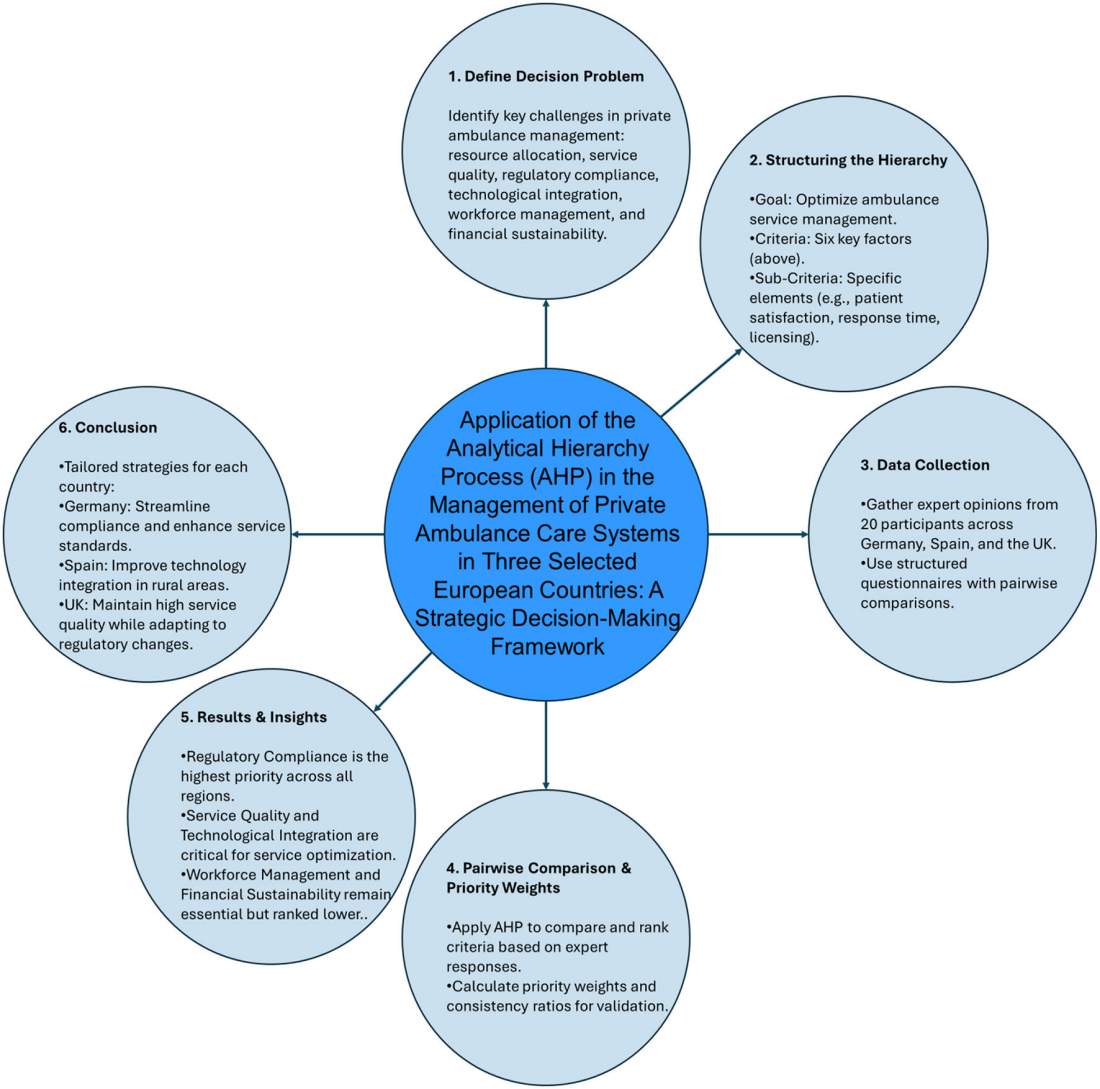
Workflow schematic for the Analytical Hierarchy Process (AHP) in private ambulance service management in Europe.

Figure 2 illustrates the systematic workflow of the Analytical Hierarchy Process (AHP) applied to private ambulance service management in Europe. The process begins with defining the decision problem, identifying key challenges such as resource allocation, service quality, and regulatory compliance. Subsequently, a hierarchical structure is established, encompassing the overarching goal of optimizing ambulance service management, the six identified criteria, and specific sub-criteria. Data collection involves gathering expert opinions from 20 participants across Germany, Spain, and the UK through structured questionnaires with pairwise comparisons. These comparisons are then used to calculate priority weights and consistency ratios, validating the data's reliability. The results and insights derived from the AHP analysis highlight regulatory compliance as the highest priority, with service quality and technological integration also deemed critical. Finally, the process culminates in tailored strategies and recommendations for each country, emphasizing streamlining compliance in Germany, enhancing technology integration in Spain, and maintaining high service quality in the UK amidst regulatory changes.

### 2.2 Application of AHP in private ambulance services across Europe

Effective management of private ambulance services is crucial for ensuring timely medical transportation, optimal resource utilization, and compliance with regulatory standards across different healthcare systems. However, these services face multifaceted challenges, including resource allocation, service quality, regulatory compliance, technological integration, workforce management, and financial sustainability. Addressing these challenges requires structured decision-making frameworks to guide service providers in optimizing operations and improving healthcare delivery. The primary objective of this study is to apply the Analytical Hierarchy Process (AHP) as a strategic decision-making tool to enhance the management and operational efficiency of private ambulance services in Europe. AHP systematically supports informed decision-making that aligns with industry needs by evaluating and prioritizing key management criteria. This study collected data from 20 participants (Germany: 7, Spain: 6, United Kingdom: 7) who are administrators, emergency medical personnel, and regulatory experts within the private ambulance sector. By structuring key challenges into a hierarchical model, AHP allows decision-makers to assess the relative importance of each criterion, leading to optimized resource allocation, improved service quality, and strengthened regulatory compliance. This study aims to develop a tailored AHP framework that identifies the most critical factors influencing ambulance service management, such as response times, cost-efficiency, and patient satisfaction and determines their relative significance. Additionally, the study explores how AHP can be applied across different European regions, considering the impact of regional regulations and healthcare system differences on ambulance service management. The findings from this research will serve as a valuable tool for private ambulance operators, healthcare administrators, and policymakers, helping them navigate operational complexities and improve service sustainability. Ultimately, this study translates the AHP-based decision-making framework into practical recommendations and strategies, contributing to more efficient, effective, and sustainable emergency medical services in Europe.

A decentralized structure with solid regional autonomy characterizes Germany's healthcare system (44). Private ambulance services are significant alongside public providers, especially in non-emergency transport and specialized medical transfers. The country's regulatory environment is complex, with regulations varying by federal states, making Germany an ideal case (45) for exploring how AHP can navigate and optimize decision-making in a fragmented regulatory landscape. In Spain, the healthcare system is largely decentralized, with each autonomous community managing healthcare services (44). Private ambulance services supplement the public system, particularly in rural and underserved areas. The challenges in Spain include navigating diverse regional regulations and ensuring service quality across geographically dispersed areas (46). Spain's mix of urban and rural settings, along with varying healthcare needs across regions (47), makes it a suitable candidate for examining the application of AHP in resource allocation and operational efficiency. The United Kingdom's National Health Service (NHS) operates a predominantly public healthcare system, but private ambulance services are often contracted to provide additional capacity, especially during peak demand or in non-emergency situations (48–50). The UK's regulatory framework for private ambulance services is stringent, strongly emphasizing quality and compliance (47). The UK's healthcare system, marked by ongoing reforms and pressures, provides a compelling context for applying AHP to enhance decision-making, particularly in service quality and regulatory compliance. The selection of these countries allows the study to examine the application of AHP across different healthcare systems and regulatory environments, providing insights into how this decision-making tool can be adapted and implemented in various European contexts.

For this study, 20 participants were selected from Germany (7 participants), Spain (6 participants), and the United Kingdom (7 participants). These participants were professionals involved in private ambulance services, including administrators, emergency medical personnel, and regulatory experts. The selection aimed to capture diverse perspectives on operational challenges, regulatory compliance, and decision-making frameworks across healthcare systems. A purposive sampling method was employed to ensure the inclusion of key decision-makers and professionals with direct experience in private ambulance services. Participants were chosen based on their expertise in emergency medical transportation, regulatory compliance, and technological integration within ambulance services. This approach enabled a focused assessment of the Analytical Hierarchy Process (AHP) application in optimizing private ambulance management. Each participant completed a structured questionnaire to collect pairwise comparisons of key decision criteria, including resource allocation, service quality, regulatory compliance, technological integration, workforce management, and financial sustainability. The responses were then analyzed using the AHP framework to derive priority weights for each criterion across the three countries.

### 2.3 Application of AHP

This study applies the AHP by demonstrating step-by-step how the AHP model is applied to a real-world decision-making scenario within the private ambulance services of the selected countries. The following steps were considered to perform the AHP framework.

° *Defining the Decision Problem:*

The primary decision problem addressed in this study is optimizing the management of private ambulance services to enhance operational efficiency, service quality, and regulatory compliance. Decision-makers must prioritize key management challenges, such as resource allocation, high service quality standards, and compliance with varying regulatory requirements.

° *Structuring the Hierarchy:*

The decision problem is structured into a hierarchical model consisting of three levels:

- **Goal**: Optimize the management of private ambulance services.
- **Criteria**: The key factors influencing the goal, which may include:

- Cost-efficiency.
- Financial Sustainability.
- Service Quality.
- Regulatory compliance.
- Technological integration.
- Workforce management.

- **Sub-Criteria**: Specific elements under each criterion, such as:

- For cost-efficiency: Operating costs, fuel consumption, and maintenance costs.
- For response time: Average response time, geographic coverage, and traffic management.
- For service quality: Patient satisfaction, training of personnel, and availability of medical equipment.
- For regulatory compliance: Adherence to local laws, cross-border regulations, and licensing.
- For technological integration: Implement GPS tracking, electronic health records, and telemedicine systems.
- For workforce management: Recruitment, retention, and staff training.

To ensure a comprehensive evaluation of the key criteria influencing the management of private ambulance services, this study employed specific sub-criteria to refine the pairwise comparison matrices. Each criterion was broken down into detailed elements that reflect its various aspects and impacts. For Resource Allocation, sub-criteria included Operating Costs, Fuel Consumption, and Maintenance Costs, which help assess the efficiency of resource distribution. Service Quality was evaluated through Patient Satisfaction, Training of Personnel, and Availability of Medical Equipment, addressing the direct and indirect factors affecting service standards. Regulatory Compliance was examined based on Adherence to Local Laws, Cross-Border Regulations, and Licensing, focusing on the complexity and scope of regulatory requirements. For Technological Integration, the study considered the implementation of GPS Tracking, Electronic Health Records, and Telemedicine Systems to gauge the extent of technology adoption and its impact. Finally, Workforce Management was analyzed through Recruitment, Retention, and Staff Training, highlighting the challenges of maintaining a skilled and stable workforce. These sub-criteria were integrated into the pairwise comparison matrices to enable a nuanced and structured analysis of each criterion's relative importance, thereby facilitating more precise decision-making in managing private ambulance services.

° *Pairwise Comparisons:*

Decision-makers from each country conduct pairwise comparisons between the criteria and sub-criteria to establish their relative importance. For instance, in Germany, regulatory compliance may be weighted more heavily due to the complex regulatory environment, while in Spain, response time may be prioritized due to the geographic diversity of the regions. These comparisons are done using a scale (e.g., 1–9), where each element is compared to another to determine which is more important and by how much.


(1) $$\begin{array}{c} A=\left[a_{ij}\right] \end{array}$$

Where *a*_*ii*_ = 1 and *a*_*ji*_ = 1/*a*_*ij*_

° *Deriving Priorities:*

The results of the pairwise comparisons are used to calculate the weights or priorities for each criterion and sub-criterion.


(2) $$\begin{array}{c} \text{Normalized matrix}:a_{ij}^{norm}=\frac{a_{ij}}{s_{j}} \end{array}$$

Where $s_{j}=\overset{n}{\underset{i=\;1}{\sum }}a_{ij}$

This involves computing the priority weights, eigenvectors, and consistency ratios to ensure the reliability of the comparisons.


(3) $$\begin{array}{c} \omega_{i}=\frac{1}{n}\sum_{j=1}^{n}a_{ij}^{norm} \end{array}$$

(4) $$\begin{array}{c} \text{Eigenvalue}:\lambda_{\max}=\;\frac{\overset{n}{\underset{i=1}{\sum }}{\left(A.\omega \right)}_{i}}{n.\omega_{i}} \end{array}$$

(5) $$\begin{array}{c} \text{Consistency Index}:CI=|\frac{\lambda_{\max}-n}{n-1}| \end{array}$$

(6) $$\begin{array}{c} \text{Consistency Ratio}:CR=\frac{CI}{RI} \end{array}$$

° *Aggregating the Results:*

The weighted criteria and sub-criteria are then aggregated to identify the most critical focus areas for private ambulance management in each country. This aggregation helps determine where resources should be allocated, and which aspects of service delivery require the most attention.

° *Decision-Making and Recommendations:*

Based on the AHP analysis, actionable recommendations are developed for each country. For Germany, the focus might be streamlining compliance processes across federal states and investing in technological solutions to enhance regulatory adherence. Improving response times through better resource distribution and upgrading technology in rural areas might be prioritized in Spain. In the United Kingdom, the recommendations could include strategies for maintaining high service quality standards while navigating the complex regulatory environment.

° *Implementation and Monitoring:*

Finally, the study outlines how these recommendations can be implemented in real-world settings, including potential challenges and strategies for overcoming them. Continuous monitoring and re-evaluation using AHP are suggested to ensure that the management of private ambulance services remains aligned with evolving healthcare needs and regulatory changes. This application of AHP provides a robust framework for addressing the diverse and complex challenges private ambulance services face in Europe. By tailoring the decision-making process to the specific context of each country, AHP enables more informed, effective, and sustainable management practices.

## 3 Results and discussion

The efficient operation of private ambulance services depends on balancing resource allocation, service quality, regulatory compliance, technological advancements, workforce management, and financial sustainability. Given the complexity of these challenges, a structured decision-making approach is essential to optimize service delivery while maintaining compliance with healthcare regulations. This study applies the Analytical Hierarchy Process (AHP) to assess and rank these factors based on their significance in Germany, Spain, and the United Kingdom, providing a data-driven perspective on key priorities for private ambulance management.

### 3.1 Pairwise comparison matrix

Effective management of private ambulance services requires a strategic approach that balances multiple operational challenges, including regulatory compliance, service quality, technological integration, workforce management, and financial sustainability. Given the complexity of these factors, a structured decision-making framework like the AHP is essential for prioritizing key criteria and optimizing service efficiency. This study applies AHP to assess and compare the management priorities of private ambulance services in Germany, Spain, and the United Kingdom. The results highlight national priorities shaped by regulatory environments, healthcare system structures, and technological capabilities. Below are each country's pairwise comparison matrices and priority weights (Tables 2–4), providing a quantitative perspective on the decision-making criteria influencing private ambulance service management.

**Table 2:** Pairwise comparison matrix representing the relative importance of key management criteria for private ambulance services in Germany, as evaluated using the Analytical Hierarchy Process (AHP).

**Criteria**	**Resource allocation**	**Service quality**	**Regulatory compliance**	**Technological integration**	**Workforce management**	**Financial sustainability**
Resource allocation	1	1/2	1/3	1/4	1/5	1/3
Service quality	2	1	1/2	1/2	1/3	1/2
Regulatory compliance	3	2	1	1/2	1/2	1/2
Technological integration	4	2	2	1	1	1/2
Workforce management	5	3	2	1	1	3/4
Financial sustainability	3	2	2	2	4/3	1

The matrix reflects expert judgments on the priority levels of resource allocation, service quality, regulatory compliance, technological integration, workforce management, and financial sustainability.

**Table 3:** A pairwise comparison matrix for Spain illustrates the relative priority of critical management criteria in private ambulance services.

**Criteria**	**Resource allocation**	**Service quality**	**Regulatory compliance**	**Technological integration**	**Workforce management**	**Financial sustainability**
Resource allocation	1	1/3	1/2	1/3	1/4	1/2
Service quality	3	1	2/3	1/2	1/2	2/3
Regulatory compliance	2	3/2	1	3/4	2/3	3/2
Technological integration	3	2	4/3	1	3/4	1
Workforce management	4	2	3/2	4/3	1	4/3
Financial sustainability	2	3/2	2/3	1	3/4	1

Using the Analytical Hierarchy Process (AHP), expert judgments evaluate the importance of regulatory compliance, technological integration, service quality, workforce management, resource allocation, and financial sustainability within Spain's decentralized healthcare context.

**Table 4:** A pairwise comparison matrix for the United Kingdom shows expert-based assessments of key decision-making criteria for managing private ambulance services.

**Criteria**	**Resource allocation**	**Service quality**	**Regulatory compliance**	**Technological integration**	**Workforce management**	**Financial sustainability**
Resource allocation	1	1/4	1/3	1/2	1/5	1/3
Service quality	4	1	2	3/2	1/3	2
Regulatory compliance	3	1/2	1	1	1/2	3/2
Technological integration	2	2/3	1	1	2/3	1/2
Workforce management	5	3	2	3/2	1	4/3
Financial sustainability	3	1/2	2/3	2	3/4	1

Through the AHP method, the table reflects how service quality, regulatory compliance, technological integration, workforce management, resource allocation, and financial sustainability are comparatively prioritized in the UK healthcare environment.

### 3.2 Priority weights calculations

Pairwise comparisons were used to calculate the priority weights for each criterion and sub-criterion in each country. The eigenvector method was applied to derive these weights, which indicate each criterion's relative importance in decision-making. Table 5 illustrates that using the AHP to the management challenges of private ambulance services in Germany, Spain, and the United Kingdom reveals distinct priorities and actionable insights for each country based on their unique contexts and needs. In Germany, the highest priority was assigned to Regulatory Compliance, with a weight of 0.25, reflecting the country's intricate and decentralized regulatory environment. This emphasizes the critical need for private ambulance services to navigate varying federal and state regulations effectively. Service Quality followed closely with a weight of 0.20, highlighting the importance of maintaining high standards across diverse regions, which is crucial given the substantial role of private providers in emergency and non-emergency contexts. Technological Integration, with a weight of 0.18, was also a key focus, underscoring the need for advanced technologies to support operational efficiency and regulatory adherence. Workforce Management (0.15) and

**Table 5:** Priority weights for Germany, Spain, and the United Kingdom.

**Criteria**	**Germany**	**Spain**	**United Kingdom**
Resource allocation	0.12	0.10	0.09
Service quality	0.20	0.18	0.22
Regulatory compliance	0.25	0.22	0.20
Technological integration	0.18	0.20	0.17
Workforce management	0.15	0.17	0.18
Financial sustainability	0.10	0.13	0.14

Financial Sustainability (0.10) were ranked lower, yet they remain significant, especially considering the high demands placed on personnel and the need to balance cost-efficiency with quality care. In Spain, the priority weights strongly emphasized Regulatory Compliance (0.22) and Technological Integration (0.20). The diverse regulatory landscape across autonomous communities and the need for technological upgrades in rural areas make these factors particularly critical. The significant weight on Service Quality (0.18) reflects the challenges of delivering consistent care across varied geographic settings. Workforce Management (0.17) and Financial Sustainability (0.13) were also important but to a lesser extent than regulatory and technological concerns. The high emphasis on technological integration highlights the necessity of investing in modern systems to bridge gaps in service delivery, particularly in underserved areas. In the United Kingdom, Service Quality emerged as the most crucial criterion with a weight of 0.22, consistent with the NHS's stringent standards and the high expectations placed on private ambulance services. Regulatory Compliance (0.20) was also critical, reflecting the complex and evolving regulatory framework that private providers must navigate. Technological Integration (0.17) and Workforce Management (0.18) were noted as important but slightly less prioritized than service quality and regulatory compliance. The focus on maintaining high service standards and ensuring compliance underscores the need for robust quality assurance programs and effective monitoring mechanisms. Financial Sustainability (0.14) was the lowest priority, although it remains essential for the long-term viability of ambulance services amid financial pressures and competition. The comparison across Germany, Spain, and the United Kingdom highlights how different regulatory environments, service demands, and technological needs shape the priorities in managing private ambulance services. Germany's focus on regulatory compliance and service quality reflects its complex federal system and high standards. Spain's emphasis on regulatory and technological integration addresses the challenges of regional diversity and technological gaps. The United Kingdom's priority on service quality and regulatory compliance aligns with its rigorous healthcare standards and regulatory expectations. These insights provide a nuanced understanding of the management challenges and suggest tailored strategies for each country to enhance the effectiveness and sustainability of private ambulance services.

**Figure 3:**
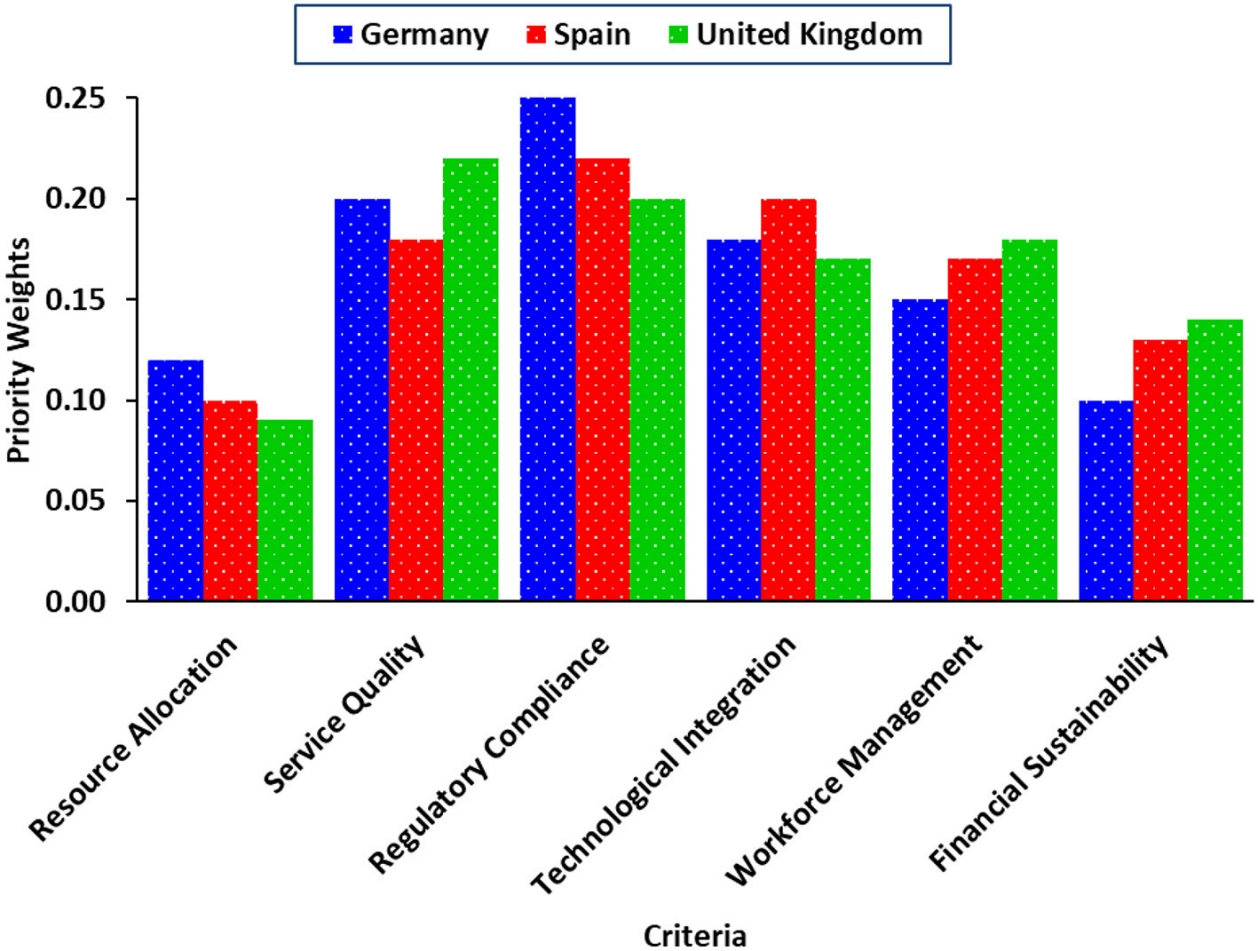
Comparative analysis of priority weights for private ambulance service criteria in Germany, Spain, and the United Kingdom.

Figure 3 presents a comparative analysis of priority weights assigned to key criteria for private ambulance services in Germany, Spain, and the United Kingdom. The results indicate that Regulatory Compliance holds the highest priority across all three countries, followed by Service Quality, which is particularly emphasized in the UK (0.22) and Germany (0.20). Technological Integration is more significant in Spain (0.20), reflecting the need for technological advancements in rural and underserved areas. Workforce Management and Financial Sustainability, though relatively lower in ranking, remain critical for operational efficiency. This visualization highlights regional differences in priority weighting, emphasizing the importance of localized decision-making strategies when managing European private ambulance services.

### 3.3 Largest eigenvalue calculation

Now, the largest eigenvalues (λ_max_) for the pairwise comparison matrices of Germany, Spain, and the United Kingdom were calculated to assess the consistency of the judgments used in the AHP. For Germany, the λ_max_ value was 6.1171, slightly above the ideal value of 6 (the number of criteria). This indicates that the judgments are reasonably consistent, but minor inconsistencies may exist. Spain's λ_max_ value was 6.0089, which is very close to 6, suggesting a high level of consistency in the judgments made. This small deviation implies that the pairwise comparisons were conducted with a strong level of coherence, leading to reliable priority weighting. The United Kingdom's λ_max_ was 5.9852, slightly below 6, which is unusual but can happen due to rounding errors or negative CI values. This result still indicates a consistent set of judgments, but the smallest eigenvalue among the three countries suggests a marginally higher consistency than in Germany (Table 6). Overall, the λ_max_ values for all three countries are close to 6, indicating that the pairwise comparison judgments were generally consistent. However, Germany shows the highest potential for inconsistency (though still within an acceptable range), while Spain and the UK demonstrate higher consistency, with Spain being the most consistent. These findings are significant as they suggest that the priority weights derived from these judgments are reliable and can be used confidently in decision-making processes in all three countries.

**Table 6:** Calculate the largest Eigenvalue λ_max_.

**Criteria**	**Germany**	**Spain**	**UK**
Resource allocation	6.2442	6.0170	5.9078
Service quality	6.1285	6.0128	5.8677
Regulatory compliance	6.0852	5.9936	5.9785
Technological integration	5.9618	6.0030	6.0527
Workforce management	6.0780	6.0053	6.0244
Financial sustainability	6.2050	6.0215	6.0800
Average λ_max_	6.1171	6.0089	5.9852

**Figure 4:**
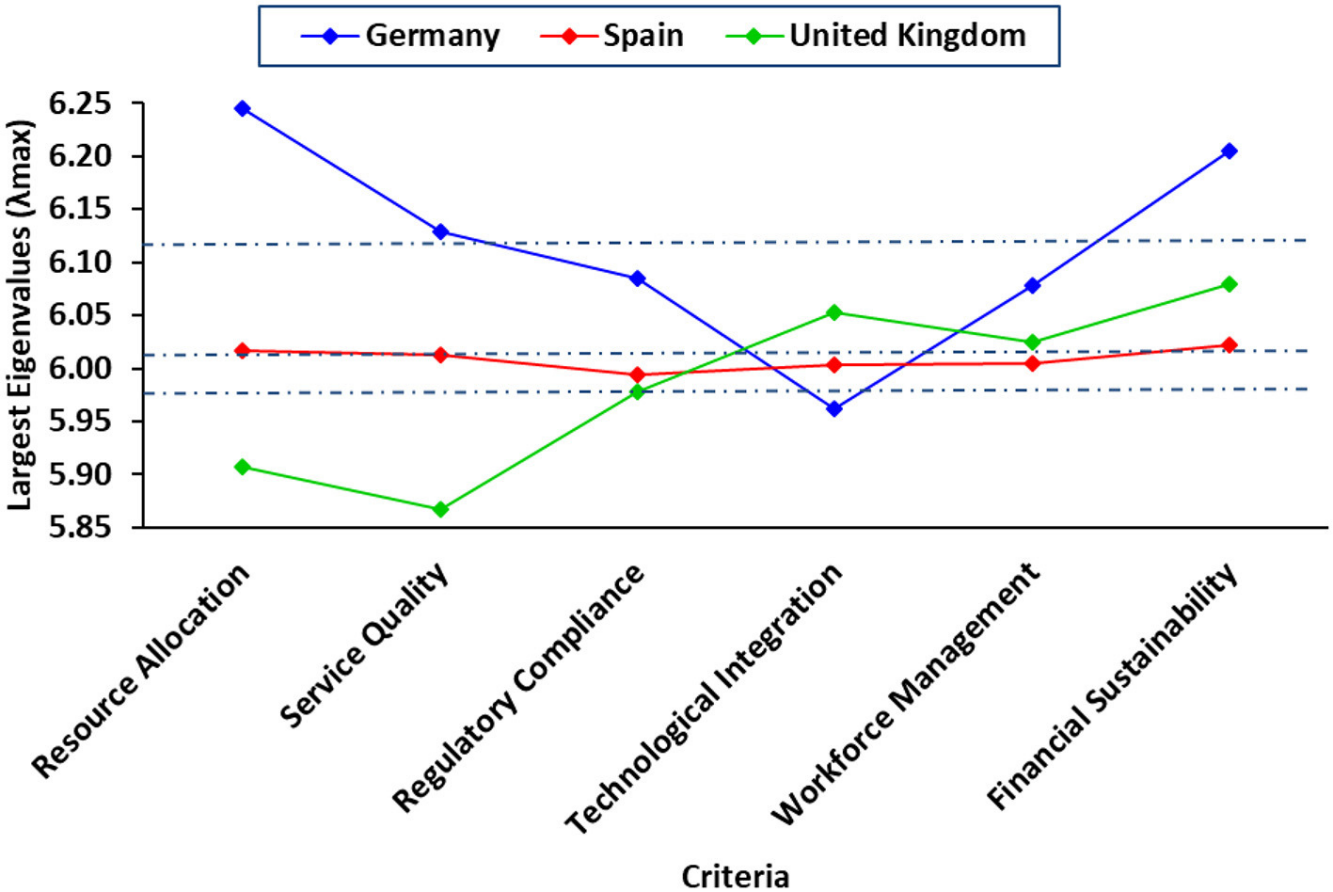
Largest Eigenvalues (λ_max_) across decision criteria for private ambulance services in Germany, Spain, and the United Kingdom, with average values indicated.

To evaluate the consistency of pairwise comparisons, the largest eigenvalues (λ_max_) were calculated for each decision criterion. Figure 4 illustrates these values for Germany, Spain, and the United Kingdom, with Spain demonstrating the highest consistency as its values are closest to 6 across all criteria. Germany exhibits slightly higher eigenvalues, suggesting minor inconsistencies in comparisons, while the United Kingdom maintains relatively stable results. The dashed horizontal lines represent the average λ_max_ values for each country, further confirming Spain's highest consistency, followed by the UK and then Germany.

### 3.4 Consistency index (CI) and consistency ratio (CR) calculations

The Consistency Index (CI) was calculated for each country to evaluate the logical coherence of the pairwise comparison judgments. For Germany, the CI was 0.0234, indicating a small degree of inconsistency. This value suggests that the pairwise comparisons are consistent but not perfectly so. In contrast, Spain's CI was significantly lower at 0.0018, reflecting a high level of consistency in the judgments. This indicates that the pairwise comparisons in Spain were highly coherent, with minimal deviations from logical consistency. The United Kingdom's CI was 0.00296, a negative value, often indicating that rounding errors or data issues may have led to an unusually low CI. Overall, Germany shows minor inconsistencies, Spain demonstrates excellent consistency, and the United Kingdom maintains reasonable coherence in its comparisons (Table 7).

**Table 7:** Calculate the consistency index (CI).

**Country**	**λ_max_**	**CI**
Germany	6.1171	0.0234
Spain	6.0089	0.0018
United Kingdom	5.9852	0.00296

The Consistency Ratio (CR) was calculated to evaluate the consistency of the pairwise comparisons relative to what would be expected from random judgments. For Germany, the CR was 0.0189, indicating that the level of inconsistency is well within acceptable limits and significantly lower than the threshold of 0.10. This suggests that Germany's pairwise comparisons are highly consistent. Spain's CR was 0.0015, which is even lower, demonstrating an exceptionally high level of consistency and confirming the reliability of Spain's comparisons. For the United Kingdom, the CR was 0.0024, which also falls well below the 0.10 threshold, indicating that despite the small negative CI, the comparisons are highly consistent. All three countries have CR values far below the critical threshold, reflecting that their pairwise comparisons are reliable and consistent (Table 8).

**Table 8:** Calculate the consistency ratio (CR).

**Country**	**CI**	**CR**
Germany	0.0234	0.0189
Spain	0.0018	0.0015
United Kingdom	0.00296	0.0024

### 3.5 Discussion

Applying the Analytical Hierarchy Process (AHP) in this study has provided a structured framework for evaluating and prioritizing key management challenges in private ambulance services across Germany, Spain, and the United Kingdom. The findings reveal that Regulatory Compliance is the most critical factor in all three countries, followed by Service Quality and Technological Integration. While Workforce Management and Financial Sustainability are relatively lower in priority, they remain essential for maintaining operational stability. The dominance of Regulatory Compliance (Germany: 0.25, Spain: 0.22, UK: 0.20) as the top priority aligns with the complex regulatory environments in European healthcare systems. Germany's decentralized healthcare model, for instance, imposes stringent regional compliance requirements, increasing the administrative burden on private ambulance operators (45). Similarly, the UK's National Health Service (NHS) regulations impose strict quality control and licensing requirements, reflecting why regulatory compliance ranks high in importance (47). Service Quality, ranking second in the UK (0.22) and Germany (0.20), aligns with (30), who emphasized cleanliness, response times, and patient satisfaction as key determinants of ambulance service quality. The relatively lower ranking in Spain (0.18) may reflect differences in public-private ambulance integration in Spain's decentralized healthcare system (44).

The emphasis on Technological Integration in Spain (0.20) suggests a growing need for advanced GPS tracking, electronic health records, and telemedicine solutions, particularly in rural and underserved areas. This finding resonates with Fatemi et al. (51), who highlighted the role of technology adoption in optimizing resource allocation and sustainability (51). Although Workforce Management and Financial Sustainability ranked lower, they remain significant. Holmberg et al. (40) emphasized that recruiting and retaining EMS personnel is a persistent challenge due to the high-stress nature of ambulance work. This challenge is especially evident in Germany and the UK, where paramedic shortages impact service delivery (40). Financial sustainability, the lowest-ranked criterion (Germany: 0.10, Spain: 0.13, UK: 0.14), suggests that while funding constraints exist, they are secondary to regulatory and service quality concerns. This contrasts with findings from Kavuma et al. (42), who identified financial constraints as a major barrier in public EMS services in Uganda. However, the difference may stem from Europe's more structured healthcare funding mechanisms (42).

The results of this study align with previous AHP applications in healthcare that emphasize the need for structured decision-making in service optimization. For instance, Schmidt et al. (33) reviewed AHP's use in healthcare and found inconsistencies in its application, highlighting the importance of a standardized approach, which this study provides (33). Similarly, Pecchia et al. (32) demonstrated that AHP effectively prioritizes key healthcare criteria, reinforcing the validity of this methodology in ambulance service management (32). Furthermore, the importance of regulatory compliance and service quality aligns with findings from Singh and Prasher (35), who combined Fuzzy AHP and SERVQUAL to assess hospital service quality. Their emphasis on patient expectations influencing quality rankings is mirrored in our study, where service quality ranks high, particularly in the UK (35). In contrast, Fatemi and Rezaei-Moghaddam (52) applied AHP in agricultural environmental management, where financial sustainability played a more dominant role. This suggests that in ambulance services, financial concerns take a backseat to regulatory and operational priorities (52).

The findings of this study have important practical implications for policymakers and ambulance service operators. First, given the high priority of regulatory compliance, private ambulance services must invest in compliance infrastructure, training, and legal expertise to navigate the complex regulatory landscape in Europe. Second, the emphasis on service quality and technological integration suggests that investment in staff training, advanced medical equipment, and digital solutions can significantly enhance ambulance service efficiency and patient outcomes. These findings align with Fager et al. (31), who found that decision-support systems improve operational efficiency in ambulance services (31). Lastly, while financial sustainability ranked lower, it remains an essential long-term consideration, particularly as healthcare funding models evolve in Europe. Strategic public-private partnerships (PPP), as explored by Gupta and Basu (39), may offer viable solutions to ensure sustainable financing models for private ambulance services.

## 4 Conclusion

Effective management of private ambulance services is crucial for ensuring timely emergency response, regulatory compliance, and high-quality patient care in diverse healthcare systems. However, these services face multifaceted challenges, requiring structured decision-making approaches to optimize resource allocation, service delivery, and financial sustainability. This study applied the Analytical Hierarchy Process (AHP) to assess and prioritize key management criteria across Germany, Spain, and the United Kingdom, offering a strategic framework for improving private ambulance service operations. The findings reveal that Regulatory Compliance holds the highest priority, with weights of 0.25 in Germany, 0.22 in Spain, and 0.20 in the UK, underscoring the significance of legal adherence and operational standardization in the private healthcare sector. Service Quality ranks second, particularly in Germany (0.20) and the UK (0.22), where patient-centered care and rapid response times are crucial. The emphasis on Technological Integration in Spain (0.20) highlights the growing need for advanced digital solutions, particularly in rural and underserved areas. While Workforce Management and Financial Sustainability rank slightly lower (Germany: 0.15 and 0.10, Spain: 0.17 and 0.13, UK: 0.18, and 0.14, respectively), they remain vital for operational efficiency and long-term sustainability. These results have important practical implications for private ambulance operators, policymakers, and healthcare administrators. Given the dominance of regulatory concerns, policymakers should focus on simplifying and standardizing compliance requirements to enhance operational efficiency while ensuring high service quality and patient safety. Additionally, investing in technological advancements such as GPS tracking, electronic health records, and telemedicine can improve efficiency, service accessibility, and overall patient outcomes, particularly in rural and underserved areas. Addressing workforce challenges through better recruitment, retention, and professional training programs is also essential for ensuring a stable and skilled workforce. Ultimately, this study demonstrates that AHP is an effective decision-making tool for prioritizing operational challenges and enhancing the efficiency of private ambulance services. By integrating structured decision-making approaches, service providers can streamline resource allocation, improve compliance with regulations, and enhance patient-centered healthcare delivery. Future research should explore the impact of evolving healthcare regulations, technological advancements, and financial sustainability models to refine strategic frameworks for private ambulance service management. Finally, the study offers tailored strategic recommendations for each country: streamlining compliance in Germany, enhancing technology integration in Spain, and maintaining high service quality in the UK amidst regulatory changes.

## Data Availability

The data analyzed in this study is subject to the following licenses/restrictions: Upon receipt of an academic inquiry, the research data related to this study can be accessed by contacting the corresponding author. Requests to access these datasets should be directed to HG; hamzehghorbani68@yahoo.com.
